# Synthesis of Lignin-Based Polyacid Catalyst and Its Utilization to Improve Water Resistance of Urea–formaldehyde Resins

**DOI:** 10.3390/polym12010175

**Published:** 2020-01-09

**Authors:** Shishuai Gao, Yupeng Liu, Chunpeng Wang, Fuxiang Chu, Feng Xu, Daihui Zhang

**Affiliations:** 1 National Engineering Laboratory for Biomass Chemical Utilization; Key Laboratory of Chemical Engineering of Forest Products, National Forestry and Grassland Administration, Key Laboratory of Biomass Energy and Material, Institute of Chemical Industry of Forest Products, Chinese Academy of Forestry, Nanjing 210042, China; gaoshishuai1006@163.com (S.G.); liuyplhs@163.com (Y.L.); wangcpg@163.com (C.W.); 2 College of Materials Science and Technology, Beijing Forestry University, Beijing 100083, China; 3 Co-Innovation Center of Efficient Processing and Utilization of Forest Resources, Nanjing Forestry University, Nanjing 210037, China

**Keywords:** lignin, catalyst, urea–formaldehyde resin, adhesive

## Abstract

In this study, a lignin-based polyacid catalyst was synthesized via two steps to enhance water resistance of urea–formaldehyde (UF) resins. The first steps involved a hydroxymethylation reaction to increase the hydroxyl content in lignin. Then, hydroxymethylated lignins were reacted with maleic anhydride to form maleated lignin-based polyacids. The acid groups were expected to function as acid catalysts to catalyze the curing process of UF resin. In order to elucidate the structural variation, 3-methoxy-4-hydroxyphenylpropane as a typical guaiacol lignin structural unit was used as a model compound to observe the hydroxymethylation and the reaction with maleic anhydride analyzed by ^1^H and ^13^C NMR. After the structural analysis of synthesized lignin-based polyacid by FTIR and ^13^C NMR, it was used to produce UF resin as an adhesive in plywood and medium density fiberboard (MDF) production, respectively. The results showed that when the addition of lignin-based polyacid was 5% in plywood, it could effectively improve the water resistance of UF resins as compared to commercial additive NH_4_Cl. It also exhibited a lower formaldehyde emission. Like plywood, lignin-based catalysts used in medium density fiberboard production could not only maintain the mechanical properties, but also inhibit the water adsorption of fiberboards.

## 1. Introduction

Urea–formaldehyde resins, generated by the polycondensation of formaldehyde, urea, and other modifiers, are one of the most important members of thermosetting resins [1,2,3]. Due to their simple synthesis process, excellent thermal properties, low curing temperature, resistance to microorganism, low cost, and excellent mechanical properties, UF resins have been widely used as wood adhesives. The total consumption of UF resins in the field of wood adhesives accounts for around 75% of formaldehyde-based resin production [3,4,5]. However, some undesirable disadvantages, such as the emission of formaldehyde and poor water resistance, have seriously hindered the wide application of UF resins. The release of formaldehyde can potentially lead to a chronic toxicity and even cancer, while the poor water resistance of resins inevitably reduces the service life of materials. Therefore, it is highly attractive to synthesize UF resins with an enhanced water resistance and acceptable formaldehyde emission [6,7,8].

In general, the polycondensation reactions that occur during the formation of UF resins are easily activated by acid catalysts to a highly crosslinked three-dimensional crystalline structure. Nevertheless, they are severely inhibited under neutral and alkaline conditions [9]. Thus, in order to promote the curing and production efficiency, acid catalysts are essential additives for UF resin in the wood-based panel industry. Currently, UF resins can be effectively catalyzed in practical production by inorganic acids or acidic salts such as ammonium chloride, ammonium sulfate, phosphoric acid, etc. [1,4]. For example, Li et al. have investigated the effects of curing agents, including ammonium chloride and hexamethylenetetramine, on the properties and performance of the UF resin [4]. The results showed that the initial viscosity, crosslinking density, and thermal stability of UF resin were enhanced by adding these curing agents. In addition, the prepress strength of UF resin was increased by 82% and 111%, respectively. However, these inorganic compounds existing in the resins tended to absorb and enrich the moisture from the air, which then resulted in the hydrolysis of UF resins [10,11]. As a result, the toxic formaldehyde is released to the environment and bonding strength is sequentially decreased. Obviously, the water resistance property and formaldehyde emission are highly relevant to the utilization of inorganic curing agents [10]. Therefore, to overcome these deficiencies, a variety of studies have recently been carried out to find alternatives to resolve the challenge in UF resin utilization. Different from inorganic acids, organic acids are generally weak acids. No acidity and water absorption are observed at room temperature. Therefore, some organic acids such as formic acid, tartaric acid, oxalic acid, and citric acid have been used as curing agents for UF resins [12,13]. For example, Nikola et al. have investigated the effect of tartaric acid addition on the curing behavior of UF resin. It showed that tartaric acid and ammonium sulfate composite catalyst system could significantly shorten the curing time of urea-formaldehyde resin without compromising the water resistance of the particleboard [13]. However, these organic acids used as curing agents of UF resins are small organic molecules, which might be vaporized during the heating process. The organic vapors can seriously affect environmental quality and human health. On the other hand, the cost of organic acids is more expensive than that of acidic salts and inorganic acids. An ideal curing agent for UF resin should be nontoxic, low-priced, and exhibit no pollution [14]. Therefore, it is still highly desirable to figure out a solution to replace organic acids without compromising other properties. Inspired by the replacement of small molecule plasticizers with macromolecules to overcome the leaking issue, we hypothesize that the addition of a polyacid catalyst can not only avoid the toxicity of organic acids, but also can effectively catalyze the curing progress to generate UF resin with enhanced water resistance. 

Currently, the utilization of renewable resources to prepare catalysts, hydrogels, and elastomers has attracted growing interest due to the sustainability concerns [15,16,17,18,19]. Being the most abundant renewable aromatic polymers, lignins are usually underutilized as a byproduct in the paper manufacturing and biorefining industries [19,20]. Only less than 2% of industry lignins are converted into high value-added chemicals. Therefore, lignin valorization has become a hot topic in academic and industrial society [21,22]. Because of the low cost, abundance, sustainability, degradable and environmentally friendly properties, lignins have been widely used as fillers, reinforcing nanoparticle and adsorbent materials [22]. Furthermore, due to various functional groups including phenolic hydroxyl, aliphatic hydroxyl, and carboxyl, the modification of lignin is feasible and has been widely performed to synthesize various interesting materials [21,23]. Therefore, we envision that lignins can potentially be used as available candidates for the preparation of bio-based polyacids. On the other hand, lignins have also been considered as promising substitutes for petroleum-based phenol to improve the properties of formaldehyde-based synthetic resins. Unfortunately, most of the active sites of lignin phenylpropane units (ortho-para positions of phenolic hydroxyl) have been occupied by methoxy groups [24]. The presence of severe steric hindrance can extremely diminish the reactivity of lignin. As compared to commercial resins, lignin-modified thermosetting resins such as lignin-phenol-formaldehyde resins and lignin-urea-formaldehyde resins require a higher temperature or more time to achieve the curing [25,26]. Therefore, it is attractive but still challenging to use lignin as fillers in UF resins to effectively improve the reactivity of lignins. 

In this study, two steps were performed to synthesize lignin-based polyacid catalysts. Firstly, in order to accurately elucidate the structural variation, 3-methoxy-4-hydroxyphenylpropane as a model compound was used to observe the hydroxymethylation and the reaction with maleic anhydride. All the structures of the model compound and its derivatives were analyzed by ^1^H and ^13^C NMR. Then, the hydroxymethylation was used to increase the hydroxyl content in lignin, which could effectively increase the reactivity of lignin not only for the subsequent modification reaction, but also help lignins to participate into the UF resin formation. Hydroxymethylated lignins were grafted with maleic anhydride to form maleated lignin-based polyacids. The structures of synthesized lignin-based polyacid catalysts were confirmed by FTIR and ^13^C NMR analysis. The acid groups were expected to function as acid catalysts to catalyze the polycondensation reaction in UF resin. The curing process, UF resin structures, thermal stability, shear strength, and formaldehyde release of UF resins with modified lignin, isophthalic acid, and NH_4_Cl were analyzed via variable temperature rheology, XRD, TGA and tensile tests, etc. The water resistance property was also investigated using plywood as the substrates. Moreover, the effect of the variation of lignin catalyst content on the mechanical properties was analyzed for medium density fiberboards, in terms of internal bond strength (IB), modulus of elasticity (MOE), and modulus of rupture (MOR). The results showed that when the addition of lignin-based polyacid was 5% in plywood, it could effectively improve the water resistance of UF resins. Moreover, a lower formaldehyde emission was observed. For medium density fiberboards, lignin-based catalysts could not only maintain the mechanical properties, but also inhibit the water adsorption of fiberboards. Due to the excellent performance of the lignin-based catalyst, it might be industrially applied in UF resin production.

## 2. Materials and Methods 

### 2.1. Materials

Analytical grade urea and formaldehyde (37%) were used for the synthesis of UF resin. Aqueous solutions of both sodium hydroxide (30%) and phosphoric acid (20%) were used to adjust the pH level during the UF resin synthesis. NH_4_Cl and maleic acid as hardeners were used. All the chemicals were purchased from Nanjing Chemical Reagents Co., Ltd. (Nanjing, Jiangsu, China). 

### 2.2. Hydroxymethylation of Lignin

A total of 20 g lignin was solubilized in 100 mL 10% aqueous NaOH, and then 20 g formaldehyde was added. The hydroxymethylation was carried out at 90 °C for 1 h. After that, the mixture was cooled to room temperature, and then acidified with 0.1 mol/L HCl aqueous. Subsequently, the precipitate (hydroxymethylated lignin) was filtered, washed to neutral, and dried in an oven at 60 °C.

### 2.3. Synthesis of Lignin-Based Polyacid Catalyst

Lignin-based polyacids were synthesized as reported by Sun et al. with minor modification [27]. First, 15 g constant weight hydroxymethylated lignin was dissolved with DMSO (50 mL) in a four-necked flask fitted with a mechanical stirrer and a reflux condenser. As a catalyst for the esterification reaction 200 μL 1-methylimidazole was added dropwise to the lignin solution, and 20 g maleic anhydride was immediately introduced into the reaction system. Subsequently, the suspension was heated to 80 °C and stirred continuously for 3 h. After that, the solution was cooled down and precipitated at pH 3 in order to recover the maleated lignin. The solid was continuously washed with water to remove the unreacted maleic anhydride. Finally, the residual solids were dried in an oven at 60 °C. The maleated lignins were named as MA-HL.

### 2.4. Model Reaction 

In order to succinctly demonstrate the formation of lignin-based polyacid catalysts, 3-methoxy-4-hydroxyphenylpropane, a compound of a typical guaiacol lignin structural unit, was used as a model compound to react with formaldehyde and maleic anhydride. The detailed process was similar to the modification of lignin. ^1^H and ^13^C NMR analysis were carried out to observe the hydroxymethylation and maleation processes.

### 2.5. Synthesis of UF Resin

UF resins with a formaldehyde/(melamine + urea) molar ratio of 1:1 were prepared in the laboratory, following a traditional alkaline–acid–alkaline technology. A total of 1200 g of formaldehyde was added once into a 2000 mL four-neck round bottom flask with a condenser, a stirring rod, and a thermometer. Subsequently, the theoretical weight of melamine and urea divided into three parts was added into the reaction system in the initial, polycondensation, and termination stages, respectively. The pH was accurately monitored and carefully adjusted via the addition of sodium hydroxide (30%) or phosphoric acid (20%). Finally, the UF resin was generated and cooled to room temperature for further utilization. 

### 2.6. Properties of UF Resins

A PB-10 acidometer (Sartorius, Göttingen, Germany) with an automatic temperature compensation was used to measure the pH value of UF resins. The UF resin viscosities were measured by a Brookfield viscometer with s61 rotor and 50 rpm spinning rate. The gel time of the UF resin with different hardeners was measured using a boiling water bath. Each curing agent was parallelly measured at least three times.

### 2.7. Preparation of Five-Plywood Panels

The urea-formaldehyde resins were mixed with 25% flour (based on UF resin total weight) and a certain amount of curing agent (ammonium chloride, maleic acid, and MA-HL) by a mechanical stirring. The five-plywood panels were made from rotary-peeled eucalyptus veneers (400 mm × 400 mm × 1.5 mm) under laboratory conditions. The adhesive blends were spread manually on the surface of eucalyptus veneers using the following conditions: 280 ± 10 g/m^2^ (double-sided) glue spreading, prepressing 1 h with 0.8 MPa prepressing pressure at room temperature, 120–125 °C hot pressing temperature, hot pressing time 60 s/mm, and 1.2 MPa hot pressing pressure.

### 2.8. MDF Panel Manufacture

All medium density fiberboards (400 mm × 400 mm × 6 mm dimension) with a density of 850 ± 20 kg/m^3^ were prepared in the laboratory. Firstly, 850 g wood fibers with a moisture content of 10–15% were weighed into a rotary blender. And then, 280 g UF resins and emulsified wax (0.5% *w*/*w* of dried fiber) were sprayed into the tank with a mechanical agitation. Subsequently, the coated fiber was dried with an aeration dryer at room temperature to decrease the initial moisture and afterwards was mixed with the required amount of lignin-based polyacids. At the end of this process, wood fibers covered with UF resin were hot-pressed with a program-controlled pressure at 170 °C for 200 s. The fiberboard was placed at room temperature for 24 h for further analysis.

### 2.9. Plywood Bonding Strength Test

The bonding strength of the plywood was determined by an electronic universal testing machine using the Chinese Standard GB/T 9846.3-2004. The testing speed was 5.0 mm/min. Test specimens for shear strength were cut into 100 mm × 25 mm pieces (gluing area of 25 mm × 25 mm). Then, in order to determine the catalytic efficiency of different curing agents, six test specimens were measured for the plywood core and 10 pieces of specimens were tested for the outer layer bonding strength. All specimens were submerged in water at 63 ± 2 °C for 3 h, and then cooled to 20 °C before the shear strength testing. 

### 2.10. MDF Mechanical and Physical Test

Mechanical properties were determined following Chinese Standard GB/T 11718-2009 using a universal testing machine. Internal bonding strength was measured in the cross-section direction at a crosshead speed of 0.5 mm/min. Modulus of rupture and modulus of elasticity of the fiberboard were obtained by performing the three-point flex test at a crosshead speed of 3 mm/min. The thickness swelling (TS) and water absorption (WA) were determined after 24 h soaking in water in accordance with GB/T 11718-2009.

### 2.11. Plywood and MDF Formaldehyde Emission Test

The formaldehyde emission from the plywood was measured by a desiccator method as described in Standard GB/T 17657-2013. The emitted formaldehyde was absorbed by 300 mL of deionized water in a 240 mm diameter container. The formaldehyde concentration of the sample solution was determined by a Shimadzu Scientific Instrument (UV-1800, Shimadzu, Kyoto City, Japan) using ammonium acetate and acetyl acetone solution method with a colorimetric detection at 412 nm. The formaldehyde emission results were the average of three times tested in parallel. Formaldehyde emission of all MDF panels was determined after seven days by a perforator method. According to this method, a 100 g specimen (about 25 mm × 25 mm × 6 mm) for each MDF panel was continually extracted for 2 h after the toluene extraction solution flowed back through the siphon tube. The formaldehyde concentration of the solution was also determined at 412 nm. The formaldehyde emission results were the average of three times tested in parallel and calculated as milligrams of formaldehyde per 100 g of dry board.

### 2.12. Characterization

The FTIR spectra of all samples were recorded using an FTIR instrument (Nicolet, USA IS10) equipped with an attenuated total reflectance (ATR) accessory. All FTIR spectra were collected at a spectrum resolution of 4 cm^−1^, with 32 co-added scans over the range from 4000 cm^−1^ to 650 cm^−1^. A background scan was acquired before scanning the samples. NMR spectra were obtained on a Bruker AVANCE3 (400 MHz) spectrometer. The chemical shifts of ^1^H and ^13^C were referenced to TMS. Thermogravimetric analysis (TGA) was performed by NETZSCH TG 209 F1 thermogravimetric analyzer. The samples were heated from 40 °C to 800 °C at a rate of 10 °C/min under a nitrogen atmosphere. A wide-angle X-ray scattering (PANalytical X’Pert Pro MPD diffraktometer) was used to investigate the crystallization of cured UF resins with different hardeners. The milled and powdered samples were analyzed at ambient temperature using a Cu Kα X-ray source (40 kV, 40 mA) with a wavelength (λ) of 1.5405 Å, in the angular range from 10° to 60° 2θ by a step of 0.02 °/s. Rheological measurements were performed on a HAAKE MARS III oscillatory rheometer with a parallel plate geometry to monitor UF resins with different curing agents. The plate diameter used was 20 mm, and the gap between the plates was 1 mm. The samples were placed between plates and sheared.

## 3. Results and Discussion

### 3.1. Model Reaction

**Figure 1:**
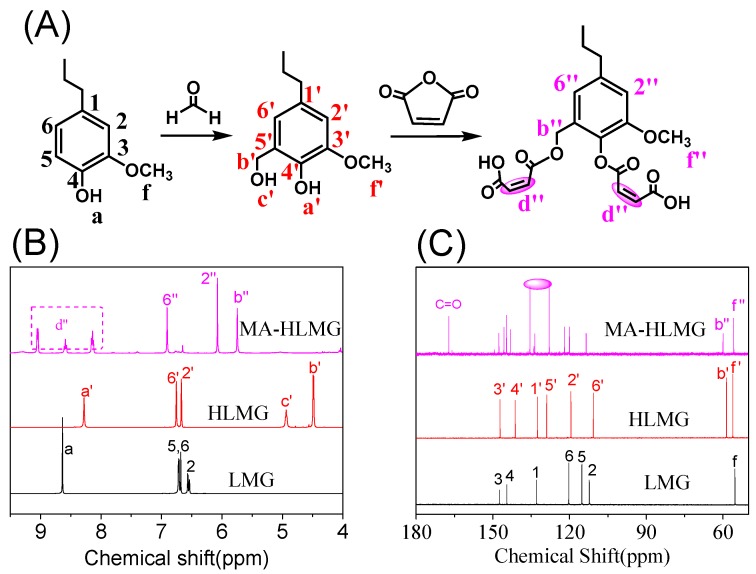
(**A**) Schematic illustration of hydroxymethylation and maleation of model compound 3-methoxy-4-hydroxyphenylpropane (LMG); (**B**) ^1^H NMR spectra of LMG and its derivatives hydroxymethylated LMG (HLMG) and maleated HLMG (MA-HLMG); (**C**) ^13^C NMR spectra of LMG and its derivatives.

Due to the complexity of lignin structures, it is difficult to directly and accurately analyze the modification process. Therefore, a model reaction using 3-methoxy-4-hydroxyphenylpropane as the model compound was firstly performed (Figure 1A). The structures of model compound and its derivatives were then analyzed by ^1^H and ^13^C NMR. After a hydroxymethlation reaction, the characteristic peaks (b’ and c’) were clearly observed, indicating the occurrence of a nucleophilic reaction between benzene rings and formaldehyde (Figure 1B). Then, the ring opening reaction of maleic anhydride and hydroxyl groups was carried out using 1-methylimidazole as the catalyst. The proton signals of hydrogel groups (b’ and c’) disappeared. As a result, the protons peaks of double bonds were shown at 8.0 to 9.1 ppm (d”). This result indicated the successful incorporation of acid groups into the model compounds. In addition, ^13^C NMR exhibited a carbon signal at 167.2 ppm, corresponding to the carbon of -COOH groups (Figure 1C), which further confirmed the occurrence of maleation reaction. 

### 3.2. Synthesis of Lignin-Based Catalysts

**Figure 2:**
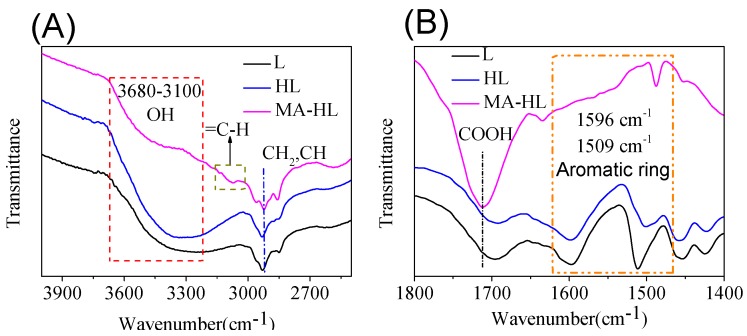
FTIR spectra in the region of 2400–4000 cm^−1^ (**A**) and 1400–1800 cm^−1^ (**B**) lignin and its derivatives, respectively (L = lignin, HL = hydroxymethylated lignin, MA-HL = maleated lignin).

After the elucidation of feasibility of reaction using a model compound, lignin was then used as a substrate to perform hydroxymethylation and to generate hydroxyl and carboxyl groups. FTIR analysis was used to observe the variation of characteristic adsorption peaks (Figure 2A,B). Obviously, after the hydroxymethylation reaction, a broad peak at 3200–3500 cm^−1^ ascribed to hydroxyl groups became more obvious due to the formation of aliphatic hydroxyl groups. It indicated the occurrence of reaction between benzene rings and formaldehyde groups [28,29,30]. In addition, when the hydroxyl groups reacted with maleic anhydride, typical characteristic peaks corresponding to the =C–H and COOH groups could be obviously observed at 3000 to 3150 cm^−1^ and 1725 cm^−1^, respectively. Furthermore, ^13^C NMR analysis also confirmed the presence of a characteristic peak (C–O) from the esterification reaction between hydroxyl groups and maleic anhydride (Figure S1). These results were consistent with the model reaction that hydroxymethylation and maleation could be simply performed under current reaction conditions. Therefore, a lignin-based polyacid catalyst was successfully prepared and used for further studies.

### 3.3. Utilization of Lignin-Based Catalyst for UF Resins Synthesis

In order to reveal the performance of the lignin-based polyacid catalyst, it was added into UF resin to catalyze the polycondensation reaction. NH_4_Cl (reacting with the free formaldehyde to release hydrochloric acid), isophthalic acid (IPA), and different amounts of polyacid catalyst were compared from the perspective of curing time. It can be seen from Table 1 that the curing rate of UF resin followed the orders: maleic acid >> ammonium chloride > isophthalic acid > blank (with no acid addition), which was positively related to the relative acidity of the curing agent. The stronger the acidity of the curing agent, the faster the curing rate of the UF resin. For the lignin-based polyacid catalyst, when its addition was 1%, the curing time was 6 min 42 sec, which was significantly higher than NH_4_Cl and IPA. However, when MA-HL amounts increased to 5%, the curing time was comparable to NH_4_Cl, indicating its potential as the acid catalyst for practical applications. 

**Figure 3:**
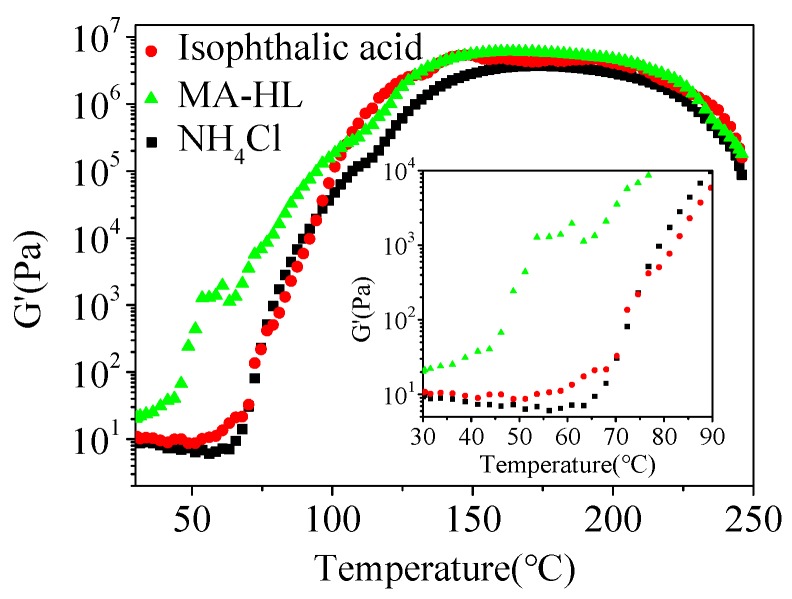
Variable temperature rheology analysis of UF resins with different additives.

The variation of viscoelastic properties versus temperature could be used as an efficient indicator to monitor the specific curing process of UF resins. Thus, we utilized the variable temperature rheology to understand the curing process. As shown in Figure 3, the curing process of IPA and NH_4_Cl was significantly different from MA-HL, particularly for the initial stage. For IPA and NH_4_Cl, firstly, as the temperature increased from 30 °C to around 60 °C, the storage modulus was slightly decreased, indicating that its viscoelasticity was mainly controlled by temperature. However, in the second stage (60 to 150 °C), the modulus quickly increased mainly due to the occurrence of polymerization to the UF resin. In addition, the evaporation of water also possibly improved storage modulus. Subsequently, a relatively flat curve was shown in the range of 150 to 200 °C, probably indicating the end of the polymerization reaction. A further increase in the temperature led to a decrease in modulus. This was due to the degradation of resin at air atmosphere. For MA-HL, its addition could enhance the initial modulus of the UF resin solution. It was important since the enhanced modulus was helpful to the practical operation and could provide a high final mechanical strength. Moreover, in the range of 30 °C to around 60 °C, the modulus remained increased, which was opposite to IPA and NH_4_Cl. This result revealed that MA-HL could catalyze the polycondensation reaction at a relatively low temperature. 

**Figure 4:**
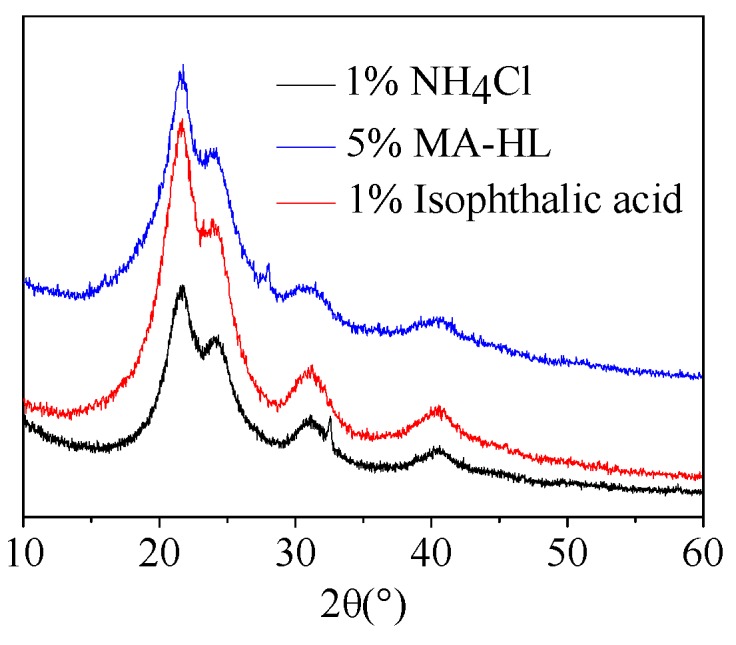
XRD patterns of UF resins with different additives.

It has been suggested that the development of UF resin crystalline regions could be harmful for the properties of UF resins [31]. Moreover, the crystalline behavior was dependent on several factors, such as the ratio of F/U, curing temperature, and hardener type [10,32]. Therefore, XRD was employed to evaluate the crystallinity variation of samples with different catalysts (Figure 4). Four obvious peaks appeared at around 2θ = 21°, 24°, 31° and 40°, respectively, indicating the presence of crystalline regions for these UF resins, which was consistent with previous studies [5,31,32]. In addition, although the intensity of signals varied slightly, different types of additives showed no significant influence on the position of signals. These results revealed that MA-HL could be used as acid catalysts in the curing of UF resin with defined crystalline structures.

**Figure 5:**
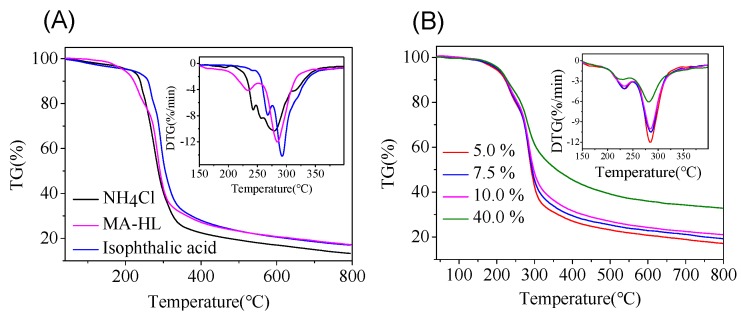
TG and DTG curves of (**A**) cured UF resins with different additives and (**B**) cured UF resins with different amounts of MA-HL.

The thermogravimetric analysis (TGA) was used to evaluate the thermal stability of the UF resin with different catalysts. It can be seen from Figure 5 and Table S1, the thermal stability varied depending on the types of additives. When 5% MA-HL was added, although the T_5%_ was slightly decreased from 213.2 °C (NH_4_Cl) and 212.5 °C (IPA) to 194.6 °C, the DTG_max_ (−12.1 %/min) was significantly reduced compared to that of IPA-catalyzed UF resin (−14.2 %/min). In addition, the UF resin cured by NH_4_Cl showed a lower initial degradation temperature. As the content of the MA-HL addition increased, the T_5%_ was slightly increased from 194.6 °C (5%) to 205.1 °C (40%), and all the T_max_ was around 280 °C. These results indicated that the thermal stability of UF resins cured by MA-HL was improved when different types of catalysts were added. In addition, it was observed that the residue was increased greatly when the addition of MA-HL was 40%. The residual was 32.8%, as compared to 17.1% (5% addition). It was possible that more lignin degradation products remained after TGA analysis.

### 3.4. Mechanical Properties Using Lignin-Based Polyacid Catalysts

**Figure 6:**
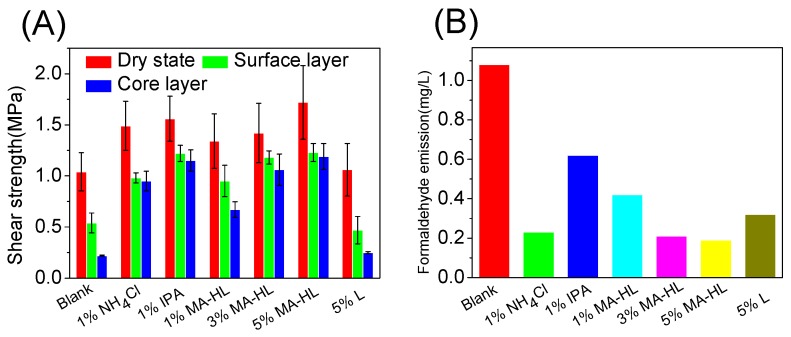
Properties of five-layer plywood prepared with UF resins containing different curing agents: (**A**) Shear strength and (**B**) Formaldehyde emission.

The mechanical properties and formaldehyde emission are two important parameters for UF resin applications. Therefore, we investigated the utilization of lignin-based polyacid catalysts on the properties of UF resins in two different substrates, plywood and medium density fiberboard. As shown in Figure 6A (plywood), the shear strengths of all substrates were improved with the addition of catalysts, as compared to blank. This was reasonable since the catalysts could be beneficial to the network formation, and thus leading to an enhanced mechanical property. In addition, plywood with 1% NH_4_Cl or 1% IPA addition showed a higher dry state shear strength (around 1.50 MPa) as compared to 1% and 3% MA-HL (1.34 and 1.42 MPa). However, when the content of MA-HL was 5%, the average shear strength in dry state could reach 1.72 MPa, which was comparable to other catalysts. In addition to the enhanced catalytic performance, the higher amounts of lignin might behave as the fillers to efficiently improve the mechanical property. On the other hand, during the hot pressing process, energy is gradually transferred from the surface layer of the plywood to the core layer, which may result in the UF resin in the core layer to be insufficiently cured. Therefore, after immersion in 63 °C water for 3 h, the bond strengths of the surface layer and core layer for plywood were also investigated to evaluate the water resistance and relative curing degree of UF resin with different curing agents, respectively. Although the wet shear strength for all the determined samples distinctly decreased, all specimens were above the minimum requirements for II grade plywood (0.7 MPa) except for the blank. Furthermore, it was observed that when the addition of MA-HL was over 3%, there was no significant variation on the shear strength of the surface and core layers, indicating the high efficiency of lignin-based polyacid catalysts. Importantly, the water resistance of plywood was elevated when 5% MA-HL was added (around 1.2 MPa), as compared to the commercial additive NH_4_Cl (around 1.0 MPa). Although MA-HL performance was similar to IPA (1.2 MPa), the non-volatile property enabled it to be a potential substitute for organic acid. Additionally, it was observed that MA-HL could significantly inhibit the formaldehyde emission when 5% MA-HL was added (0.12 mg/L, E0 grade 0.5 mg/L) in comparison to IPA (0.6 mg/mL, E1 grade 1.5 mg/L, Figure 6B). It was believed that the remaining reactive sites in lignin could potentially react with formaldehyde to decrease its emission. Finally, the unmodified lignin (5%) was also added into the UF resin to make a comparison. The results showed that the strength of the surface layer with lignin addition (0.47 MPa) was even slightly lower than the blank (0.54 MPa) due to the aggregation of lignin, while the one with modified lignin (5%) as catalyst can reach 1.23 MPa. These comparisons further supported our conclusion that a lignin-based polyacid catalyst can significantly improve the water resistance of UF resins.

**Figure 7:**
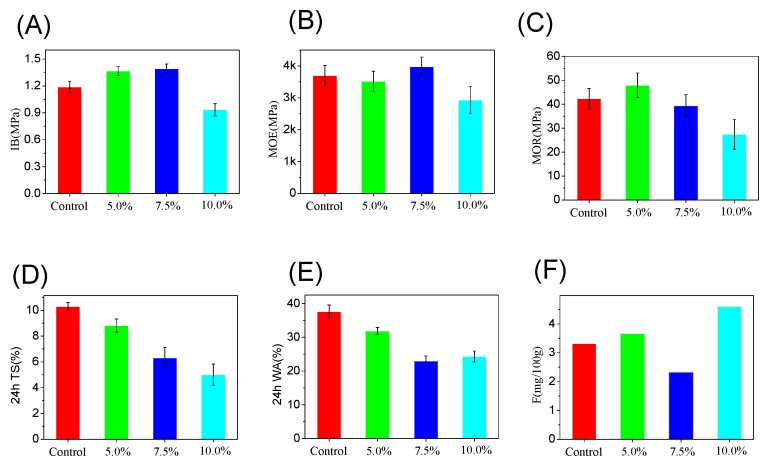
Mechanical properties of medium density fiberboard. (**A**) Internal bond strength (IB), (**B**) modulus of elasticity (MOE), (**C**) modulus of rupture (MOR), (**D**) 24-h thickness swelling rate, (**E**) water adsorption of fiberboard, (**F**) formaldehyde emission of medium density fiberboard.

For medium density fiberboards, mechanical properties in terms of internal bond strength (IB), modulus of elasticity (MOE), and modulus of rupture (MOR) have been systematically investigated (Figure 7A–C). When 5% MA-HL was added, its IB, MOE, and MOR were comparable to the control (1% NH_4_Cl addition), indicating its potential use in the production of medium density fiberboards. In addition, as the content of MA-HL increased, we found that its effect on the mechanical properties was different. For example, when 7.5% MA-HL was used, it could slightly increase the IB and MOE, whereas the MOR was decreased. When the content of MA-HL was further increased to 10%, it was detrimental to the mechanical properties. This could be due to the poor dispersion of lignin. The water adsorption analysis was further determined to evaluate the water resistance properties. As shown in Figure 7D,E, the addition of lignin-based polyacid catalysts could effectively decrease the water adoption in terms of volume and weight variation. This might be due to the hydrophobic property of lignin, which increased the water resistance of UF resin. For the formaldehyde emission, MA-HL addition (5% or 10%) slightly increased its emission as compared to NH_4_Cl, but it still met the formaldehyde emission requirements of E0 grade fiberboard (5 mg/100 g). Moreover, for 7.5% MA-HL addition, the formaldehyde emission is 2.32 mg/100 g, which below the minimum level of Japanese F **** grade standard (3 mg/100 g).

## 4. Conclusions

In this study, a lignin-based polyacid catalyst was synthesized via two steps to enhance the water resistance of UF resins. Both hydroxymethylation and maleation reactions could effectively occur to introduce acid and hydroxyl groups, which was confirmed by a model reaction and the structural analysis of modified lignin. The lignin-based polyacid catalyst could be used as an efficient catalyst to promote the polycondensation reaction in UF resin formation. When the synthesized UF resin was used as the adhesive in plywood and medium density fiberboard production, the utilization of the lignin-based catalyst could effectively improve the water resistance of UF resins as compared to commercial additive NH_4_Cl. Moreover, in plywood preparation, it exhibited a lower formaldehyde emission. For medium density fiberboards, lignin-based catalysts used in UF resins could not only maintain the mechanical properties, but also inhibit the water adsorption of fiberboards, indicating their potential to replace small molecule curing agents in the UF resins fields.

## Figures and Tables

**Table 1:** Curing time, shear strength, and formaldehyde release of UF resins with different curing agents.

Curing Agent Species	Curing Time	Shear Strength/MPa			Formaldehyde Emission/mg/L
		Dry State	Surface Layer	Core Layer	
Blank	>15 min	1.04	0.54	0.22	1.08
1% NH_4_Cl	2 min 45 s	1.49	0.98	0.95	0.23
1% IPA	3 min 10 s	1.56	1.22	1.15	0.62
1% MA-HL	6 min 42 s	1.34	0.95	0.67	0.42
3% MA-HL	3 min 56 s	1.42	1.18	1.06	0.21
5% MA-HL	2 min 50 s	1.72	1.23	1.19	0.19
5% L	>15 min	1.06	0.47	0.25	0.32
Maleic acid	Gelling at room temperature in a few minutes				

## Supplementary Materials

The following are available online at https://www.mdpi.com/2073-4360/12/1/175/s1, Table S1: TG and DTG results of cured UF resins with different additives. ^13^C NMR Spectra of lignin and modified lignin; Figure S1 ^13^C NMR spectra of lignin and modified lignin.
