# Supplementary material for: Synthesis of Lignin-Based Polyacid Catalyst and Its Utilization to Improve Water Resistance of Urea–formaldehyde Resins
Source: Polymers (Basel). 2020 Jan 9;12(1):175. doi: 10.3390/polym12010175 (PMC7022909; doi:10.3390/polym12010175)
Supplement: Supplementary file 1 [file polymers-12-00175-s001.zip › polymers-678115-SI.docx]

Synthesis of Lignin Based Polyacids Catalyst and Its Utilization to Improve Water Resistance of Urea Formaldehyde Resins

**Shishuai Gao^1,2,3^, Yupeng Liu^1,3^, Chunpeng Wang^1,3^, Fuxiang Chu^1,3*^, Feng Xu^2*^,Daihui Zhang^1,3*^**

^1^ Institute of Chemical Industry of Forest Products, Chinese Academy of Forestry; National Engineering Laboratory for Biomass Chemical Utilization; Key Laboratory of Chemical Engineering of Forest Products, National Forestry and Grassland Administration; Key Laboratory of Biomass Energy and Material; Nanjing, 210042, Jiangsu, China; gaoshishuai1006@163.com(S.G.); liuyplhs@163.com(Y.G.); wangcpg@163.com(C.W.); chufuxiang@caf.ac.cn(F.C.); zdh0824@163.com(D.Z.)

^2^ College of Materials Science and Technology, Beijing Forestry University, Beijing 100083, China; gaoshishuai1006@163.com(S.G.); xfx315@bjfu.edu.cn(F.X.)

^3^ Co-Innovation Center of Efficient Processing and Utilization of Forest Resources, Nanjing Forestry University, Nanjing, 210037, Jiangsu, China; gaoshishuai1006@163.com(S.G.); liuyplhs@163.com(Y.G.); wangcpg@163.com(C.W.); chufuxiang@caf.ac.cn(F.C.); zdh0824@163.com(D.Z.)

***** Correspondence: chufuxiang@caf.ac.cn(F.C.); xfx315@bjfu.edu.cn(F.X.); zdh0824@163.com(D.Z.); Tel.: +8685482473 (D.Z.)

**Table S1** TG and DTG results of cured UF resins with different additives.

| **Hardeners** | **T_5%_ (^o^C)** | **T_max_ (^o^C)** | **DTG_max_ (%/min)** | **R (%)** |
| --- | --- | --- | --- | --- |
| 1% NH_4_Cl | 213.2 | 281.7 | -10.3 | 13.3 |
| 1% IPA | 212.5 | 294.5 | -14.2 | 17.0 |
| 5% MA-HL | 194.6 | 284.6 | -12.1 | 17.1 |
| 7.5% MA-HL | 201.8 | 285.9 | -10.5 | 19.2 |
| 10% MA-HL | 204.3 | 284.8 | -10.1 | 21.0 |
| 40% MA-HL | 205.1 | 281.3 | -6.1 | 32.8 |

**Figure S1** ^13^C NMR Spectra of Lignin and Modified Lignin
